# Genetics of CAKUT

**DOI:** 10.1515/medgen-2025-2053

**Published:** 2026-02-18

**Authors:** Alina C. Hilger, Rik Westland, Julia Hoefele

**Affiliations:** University Hospital Erlangen Department of Pediatrics and Adolescent Medicine, Department of Pediatric Nephrology Loschgestr. 15 91054 Erlangen Germany; Department of Pediatric Nephrology Amsterdam UMC location University of Amsterdam Meibergdreef 9 1105 AZ Amsterdam The Netherlands; Institute of Human Genetics University Hospital Ludwig-Maximilians University Goethestr. 29 80336 Munich Germany

**Keywords:** CAKUT, extrarenal manifestations, syndromic CAKUT, dominant inheritance, polygenic burden

## Abstract

Congenital anomalies of the kidney and urinary tract (CAKUT) represent a heterogeneous group of developmental disorders and are the leading cause of pediatric chronic kidney disease worldwide. The phenotypic spectrum is broad, encompassing kidney agenesis, hypodysplasia, multicystic dysplastic kidneys, vesicoureteral reflux, obstructive uropathies, and other malformations affecting the kidneys, ureters, and urethra. Advances in genetics have begun to unravel the molecular pathways underlying these diverse phenotypes, yet the complexity of CAKUT reflects contributions from both monogenic variants and multifactorial causes.

This review provides an overview of the current understanding of the genetic causes of CAKUT, beginning with fundamental principles of kidney and urinary tract development, and then focusing on major discoveries in the past ten years. We aim to summarize key genetic findings, with an emphasis on genotype–phenotype correlations and developmental pathways, highlight emerging mechanisms, and discuss their implications for diagnosis, counseling, and clinical management.

## Introduction

Congenital anomalies of the kidney and urinary tract (CAKUT) are among the most common developmental disorders, accounting for a substantial proportion of pediatric chronic kidney disease and contributing significantly to morbidity in adulthood [1]. The spectrum of CAKUT is broad and can be systematically categorized according to the anatomical site of the defect:

Kidney anomalies: duplex kidney, kidney agenesis, hypoplasia, dysplasia, and multicystic dysplastic kidney.
Ureteral anomalies: duplex collecting systems, megaureter, ureteropelvic junction obstruction, ureterovesical junction obstruction, vesicoureteral reflux and ectopic ureters.
Urethral anomalies: posterior urethral valves, urethral atresia, congenital urethral stenosis, and other forms of lower urinary tract obstruction.

Congenital defects of the bladder, such as extropic bladder or non-neurogenic neurogenic bladder, are generally not considered to belong to the CAKUT spectrum.

As CAKUT leads to reduced kidney mass and subsequent lower nephron number, these children are by definition at risk for hypertension, proteinuria and reduced glomerular filtration rate later in life [2, 3]. However, as nephron number cannot be counted in vivo, current guidelines recommend that all children with reduced general mass reduction should be followed on a regular basis [4, 5]. Better understanding of the genetic etiology of CAKUT may provide the basis for devising individualized clinical management for individuals with CAKUT by defining at risk and not at risk patient.

However, the etiology of CAKUT is heterogenous. Over 50 monogenic contributors have been implicated in syndromic CAKUT (i.e. a CAKUT phenotype in conjunction with extrarenal malformations) as well as isolated CAKUT cases, often involving genes essential for key developmental pathways or transcription factors [6]. Clinical variability even within families carrying the same variant, incomplete penetrance, and variable expressivity complicate clear genotype–phenotype correlations [7, 8].

In the past ten years, rapid advances in genetic technologies – especially towards genome sequencing, genome-wide association studies, and functional modeling using animal models as well as human kidney organoid systems – have markedly expanded the understanding of the underlying genetic (and non-genetic) pathomechanisms leading to the development of CAKUT [8–10]. These efforts have identified new disease-associated genes, elucidated pathogenic mechanisms within key developmental pathways (e.g., transcriptional regulation, signaling networks, extracellular matrix interactions), and revealed the contribution of noncoding variation and epigenetic regulation [11, 12].

In this review, we summarize the current understanding of the genetic basis of CAKUT. Given the breadth of CAKUT genetics, this review primarily focuses on molecular discoveries and their clinical implications, rather than an exhaustive account of all developmental pathways. After beginning with pivotal fundamental developmental principles, we focus on highlighting key advances reported in the past decade. We also discuss their implications for diagnosis, counselling, and clinical management.

## Fundamentals of CAKUT genetics

The development of the kidney and urinary tract is a highly coordinated process that relies on reciprocal interactions between the ureteric bud and the metanephric mesenchyme [13]. Early genetic studies of CAKUT provided the foundation for understanding how developmental perturbations of the kidney and urinary tract result in congenital kidney anomalies. These studies identified a core set of monogenic causes, many of which involve transcription factors and signaling pathways such as GDNF/RET and WNT, essential for kidney and urinary tract development. They regulate nephron induction, ureteric bud branching, and subsequent urinary tract morphogenesis. Hence many well-established human CAKUT genes but also candidate genes derived from studying murine nephrogenesis can be found in these pathways and include key transcription factors (*PAX2, EYA1, SIX1, GATA3*), *GDNF/RET* Signaling Pathway [*RET*, *ITGA8, GDNF (identified in mice)* *GFRA1*], WNT Signaling Pathway [*WNT4, WNT9B (identified in mice), FZD8 (identified in mice) LRP4*], BMP and FGF Signaling Pathways (*BMP4, BMP7, FGF20, FGFR2*). Table 1 provides a selection of validated monogenic causes of CAKUT, while Table 2 summarizes emerging candidate genes and genomic mechanisms.

Disruption of these pathways, with pleiotropic roles in embryogenesis can result in a wide spectrum of kidney anomalies. Further, it can be observed that variants within those crucial developmental pathways often lead to syndromic forms of CAKUT.

## Diagnostic yield in CAKUT genetics

These canonical developmental pathways laid the foundation upon which recent genome-wide and multi-omics discoveries have expanded our understanding of CAKUT pathogenesis. Nevertheless, the genetic basis is currently solved in only a minority (<20 %) of cases from unselected cohorts. However, this primary set of identified CAKUT genes laid the ground for a new era of discovery. Enabled by next-generation sequencing including genome sequencing, large-scale genomic studies, and novel functional modeling systems, the knowledge of CAKUT genetics has dramatically expanded in recent years. Further, animal models, particularly mice, have been instrumental in identifying critical developmental regulators, while more recently, human pluripotent stem cell–derived kidney organoids have provided a platform to study nephrogenesis and disease modeling in a human-specific context [9].

**Table 1:** Selection of established key genes and copy number variations associated with CAKUT. ECM, extracellular matrix.

Category	Representative genes
Transcription factors	*PAX2, EYA1, SIX1, SIX2, SALL1, HNF1B, PBX1, GATA3, WT1, LHX1*
Signaling pathways	*RET, GDNF, GFRA1, ITGA8, BMP4, BMP7, FGF20, FGFR2, WNT4, WNT9B, LRP4*
Structural / ECM	*FRAS1, FREM1, FREM2, GRIP1, COL4A1-A5*
Ciliopathy / polarity	*NPHP1, NPHP3, CEP290, VANGL1, VANGL2, WDPCP*
Miscellaneous	*TBX18, FOXC1, NR6A1,*
Copy-number variants	Chr17q12 (*HNF1B*), Chr22q11.2 (*CRKL*), Chr 16p11.2 (*TBX6/MAZ*), Chr1q21.1

## Recent advances in CAKUT genetics – from gene discovery to mechanism

Over the past ten years, major advances in genomic technologies and functional modeling have substantially broadened our understanding of CAKUT genetics, adding up to the previously identified and mentioned disease genes. Below, we give several recent key examples how genetic discoveries have extended our understanding of human kidney (mal)development (Table 2):

### Novel monogenic discoveries

*ZMYM2* (chr13q12.11): *De novo* or inherited heterozygous loss-of-function variants cause syndromic CAKUT. Reported kidney/urinary tract phenotypes include kidney agenesis, kidney hypodysplasia, dysplasia, hydroureter, duplex collecting systems, and vesicoureteral reflux. Extrarenal manifestations comprise craniofacial dysmorphism, congenital heart defects, small hands and feet with dysplastic/hypoplastic nails, clinodactyly, and neurological features [14].
*FOXC1* (chr6p25.3): Originally linked to Axenfeld–Rieger syndrome, but recent studies expanded its phenotypic spectrum to include CAKUT. Kidney findings include bilateral hypodysplasia, renal cysts, vesicoureteral reflux, and lower urinary tract obstruction with obstructive uropathy. Extrarenal features often include anterior segment dysgenesis, glaucoma, and hearing loss. [15]
*FOXD2* (chr1p32.2): Homozygous loss-of-function variants were recently shown to cause syndromic CAKUT with bilateral renal pelvic dilation, papillary atrophy, and vesicoureteral reflux. Reported extrarenal features include microcephaly, growth restriction, and developmental delay [16]
*BNC2* (chr9p22.3): Heterozygous loss-of-function and missense variants in *BNC2* were identified in families with congenital lower urinary tract obstruction (LUTO) and other, likely secondary, CAKUT phenotypes. Reported renal and urinary tract findings include posterior urethral valves, urethral obstruction, hydronephrosis and hydroureter. *BNC2* encodes a zinc-finger transcription factor expressed in the ureteric mesenchyme and urothelium [17].
*TSHZ3* (chr19q13.11): Heterozygous variants or deletions affecting *TSHZ3* have been associated particularly with multicystic dysplastic kidney, hydronephrosis, and hydroureter, and, inconsistently, with specific extrarenal features, including genital anomalies. The gene encodes a transcription factor regulating smooth muscle cell differentiation via interaction with SRY-box transcription factor 9 (*SOX9*) and myocardin (*MYOCD*) during ureter (and kidney) development [18].
*ARID3A* (chr19p13.3): *De novo* heterozygous variants in *ARID3A* were reported in patients with syndromic CAKUT showing kidney hypodysplasia, cystic dysplasia, and vesicoureteral reflux, sometimes accompanied by developmental delay and craniofacial features. *ARID3A* encodes a transcriptional regulator influencing nephron progenitor differentiation and Wnt-signaling dynamics [9].
*NR6A1* (chr9q33.3): Heterozygous variants in *NR6A1* have been linked to CAKUT phenotypes such as unilateral or bilateral kidney hypodysplasia, vesicoureteral reflux, and ureteral obstruction. NR6A1 (Nuclear Receptor Subfamily 6 Group A Member 1) acts as a developmental transcriptional regulator required for urogenital ridge patterning [19].
*GFRα1* (chr10q26.11): Biallelic or heterozygous pathogenic variants in *GFRα1* (GDNF Family Receptor α1) have been identified in individuals with syndromic and nonsyndromic CAKUT. Reported renal and urinary tract manifestations include kidney agenesis, hypodysplasia, and vesicoureteral reflux. GFRα1 functions as a co-receptor for GDNF signaling through RET, essential for ureteric bud outgrowth and branching morphogenesis [20].

### Insights from large-scale genomic studies

Given the heterogeneity of CAKUT genes and the still existing paucity of knowledge about potentially underlying genetic causes in up to 80 % of cases, GWAS have revealed additional contributors to CAKUT risk.

Planar cell polarity and WNT pathways: a large case–control GWAS combined with CNV (copy number variations) analysis of vesicoureteral reflux (VUR) identified study-wide significant and suggestive loci that map to genes involved in developmental signalling, including *WDPCP* (chr2p15), *VANGL1* (chr1p13.1) and *WNT5A* (chr3p14.3), and follow-up functional studies in *Wnt5a* mutant mice supported a role for WNT5A in bladder and ureteric morphogenesis and ureteral insertion [21].
Lower urinary tract obstruction: Two complementary studies from van der Zanden et al. and Chan et al investigated posterior urethral valves. The study from van der Zanden represents the largest array-based GWAS meta-analysis to date but lacked genome-wide significant hits and reported three suggestive loci (chr13q21.32, 16q12.1, 20q13.31), reflecting limited power in a rare condition. Chan et al. used WGS and a trans-ancestry approach and was able to nominate *TBX5* and *PTK7* as PUV susceptibility genes while also implicating structural/regulatory variation. Together these studies point toward involvement of developmental transcriptional regulators and planar-cell-polarity / morphogenesis pathways, but larger cohorts and multi-modal data are still required to fully define the role of these loci in PUV genetics [22, 23].

These studies suggest that CAKUT arises from both rare monogenic variants and a polygenic burden of developmental alleles, implicating shared developmental pathways.

### Structural variants and non-coding mechanisms

Beyond single nucleotide variants, structural variants and non-coding variants contribute substantially to CAKUT. Recurrent CNVs explain a measurable fraction (~3–6 %) of cases in population series and often flag syndromic comorbidity (neurocognitive, cardiac).

Recurrent CNVs (Table 2):
  17q12 deletions (including *HNF1B*) → renal cystic dysplasia, hypodysplasia, agenesis; the extrarenal spectrum is extensive and includes diabetes mellitus, pancreatic atrophy as well as developmental delay, autism spectrum disorder and hypomagnesemia and hyperuricaemia [24].
  22q11.2 deletions → hydronephrosis, kidney agenesis, duplex systems; often in the context of DiGeorge/velocardiofacial syndrome. Additional clinical findings may include cardiac and gastrointestinal malformations, hypocalcemia, hearing loss and skeletal anomalies [24],
  16p11.2 and 1q21.1 deletions → variable kidney phenotypes including vesicoureteral reflux and kidney agenesis; also associated with neurodevelopmental disorders [24]. 16p11.2 microdeletion syndrome is associated with autism spectrum disorder, obesity and scoliosis, whereas 1q21.1 microdeletions may include developmental delay, facial dysmorphic features and eye abnormalities.
Non-Coding and Regulatory Variants: Single-cell multi-omics analyses of fetal kidney and organoid models, such as a single-cell multi-omics analysis of kidney organoid differentiation, have begun to map enhancers and cis-regulatory elements essential for nephrogenesis. Disruption of such regions is now recognized as a possible mechanism for CAKUT in patients without coding variants. Nevertheless evidence from human data is still missing [25].

### Functional modeling and translational insights

Functional studies remain crucial to establish causality and mechanism. Functional validation has shifted from single-gene mouse knockouts toward integrative models (Xenopus or zebrafish rapid screens, human iPSC/kidney-organoid assays, and organoid single-cell multi-omics). These platforms have been used to validate causality and elucidate pathways. Hence, besides newly identified CAKUT genes (*ZMYM2*, *FOXD2*) also well-established CAKUT Genes (*HNF1B*) are recently studied:

*ZMYM2*: Xenopus knockdown and *Zmym2*^+/−^ mouse models reproduced kidney agenesis, dysplasia, and urinary tract anomalies, confirming its role in kidney development [14].
*FOXD2*: Knockout mice showed papillary defects, renal pelvic dilation, and impaired ureteric bud branching, consistent with human phenotypes [16].
*HNF1B*: Human iPSC-derived kidney organoids with engineered *HNF1B* variants revealed tubular morphogenesis defects and downstream transcriptional dysregulation, demonstrating the value of organoid systems for human-specific disease modeling [9].

Together, these platforms allow mechanistic dissection of CAKUT genes across species and might provide models for future testing of therapeutic strategies.

## Clinical implications

The clinical hallmarks of CAKUT of variable expressivity, incomplete penetrance and the relatively low diagnostic yield for monogenic causes of CAKUT have impacted the indication for genetic testing in the clinical setting. Clinical recommendations as defined by the ERA working group for genetic diseases to perform genetic testing in CAKUT have been limited to familial occurrence, the presence of extrarenal abnormalities or a suspicion for a syndromic disorder as well as more “soft” indications such as ending diagnostic odysseys and to counsel future pregnancies.^32^ Recent studies also show that genes previously described only in a syndromic context can lead to isolated CAKUT phenotypes, depending on where the corresponding variant is located [27, 28]. This particularity makes genetic counseling for these families essential. In addition, each new variant must also be assessed with regard to its location in the respective gene. Large-scale genomic sequencing studies can help to generate better genotype-phenotype correlations in the future, not only for individual genes but also for individual variants. It is highly recommended to involve a clinical geneticist when the decision to perform genetic testing has been made to aid in the interpretation and counseling of patients and their family members. To highlight the importance of genetic counseling and molecular genetic testing finding the correct diagnosis, the following two case reports would like to be given:

First, we would like to report on a 30-year-old male patient who, due to a family history of diabetes mellitus, underwent testing at the age of 25 years and was diagnosed with type II diabetes mellitus. Five years later, he was already insulin-dependent, and the diagnosis of type II diabetes mellitus was maintained.

During a routine abdominal ultrasound examination at the age of 30 years, bilateral renal hypoplasia was detected. The renal specialists suspected an autosomal dominant tubulointerstitial kidney disease (ADTKD). During human genetic counseling, however, suspicion of *HNF1B*-associated nephropathy (Renal Cysts and Diabetes syndrome, RCAD) was raised, which was confirmed by molecular genetic testing (detection of a heterozygous pathogenic variant in *HNF1B*) explaining both, the renal hypoplasia as well as the diabetes.

**Table 2:** Emerging candidate genes associated with CAKUT

Gene/Locus	Chromosome	Renal/Urinary Tract Phenotypes	Extrarenal Phenotypes	Syndrome/Context	First Report (Author, Journal, Year)
***ZMYM2***	13q12.11	Kidney agenesis, hypodysplasia, dysplasia, duplex kidneys, hydroureter, vesicoureteral reflux	Craniofacial dysmorphism, neurodevelopmental delay, cardiac defects	Syndromic CAKUT	Connaughton et al., *Am J Hum Genet*, 2020 [14]
***GFRA1***	10q26.11	Kidney agenesis, kidney dysplasia	-	Isolated CAKUT	Arora et al., *J Am Soc Nephrol.,* 2021 [20]
***FOXC1***	6p25.3	Kidney hypodysplasia, cystic kidneys, vesicoureteral reflux, obstructive uropathy	Ocular anomalies (Axenfeld–Rieger), hearing loss, glaucoma	Expanded Axenfeld–Rieger phenotype incl. CAKUT	Wu et al., *Genet Med*, 2020 [15]
***FOXD2***	1p32.2	Bilateral renal pelvic dilation, papillary atrophy, VUR	Microcephaly, growth restriction, developmental delay	Syndromic CAKUT	Riedhammer et al., *Kidney Int*, 2024 [16]
***BNC2***	chr9p22.3	Posterior urethral valves, urethral obstruction, hydronephrosis and hydroureter	-	Isolated CAKUT	Kolvenbach et al., *Am J Hum Genet,* 2019 [17]
***ARID3A***	19p13.3	Kidney hypodysplasia, cystic dysplasia, and vesicoureteral reflux	Developmental delay and craniofacial features	Syndromic CAKUT	Rasouly et al., *Nat. Commun.* 2025 [19]
***NR6A1***	9q33.3	Unilateral or bilateral kidney hypodysplasia, vesicoureteral reflux, and ureteral obstruction	-	Isolated CAKUT	Rasouly et al., *Nat. Commun.* 2025 [19]
***TSHZ3***	19q13.11	Multicystic dysplastic kidney, hydronephrosis, and hydroureter	Genital anomalies	Syndromic CAKUT	Kesdiren et al., *Eur J Hum Genet*, 2025 [18]
***WDPCP***	2p15	Vesicoureteral reflux, hydronephrosis	-	Implicated in PCP pathway	Verbitsky et al., *J Am Soc Nephrol*, 2021 [21]
***VANGL1***	1p13.1	Vesicoureteral reflux, kidney hypodysplasia	-	PCP pathway gene	Verbitsky et al., *J Am Soc Nephrol*, 2021 [21]
***WNT5A***	3p14.3	Vesicoureteral reflux, obstructive uropathy	-	WNT signaling	Verbitsky et al., *J Am Soc Nephrol*, 2021 [21]

The patient’s father has also had diabetes mellitus since the age of 24 years. Two uncles on his father’s side have insulin-dependent diabetes mellitus and have already had kidney transplants. His paternal grandmother had insulin-dependent diabetes mellitus and died at the age of 50 years. Although segregation could not be performed in this family, it is very likely that the disease-causing variant was inherited from the father resp. the paternal side, which also explains the clinical symptoms of the index patient’s relatives.

Second, we would like to present a family with a kidney disease that has remained unclear for decades: the index case was the 6-year-old daughter, who was diagnosed with proteinuria. An ultrasound of her kidneys revealed bilateral kidney hypoplasia. The patient’s father had been under nephrological care for over a decade with CKDx (chronic kidney disease of unknown origin). Molecular genetic diagnostics had not yet been performed.

During human genetic counseling, which was initiated by the family and not by the medical colleagues, minor dysmorphic features were detected concerning the ears (condition after preauricular skin tag) and hands (triphalangeal thumbs on both sides) of the father and daughter. In addition, the father now had hearing impairment and anal canal stenosis as an infant, which was treated surgically. Molecular genetic testing revealed a heterozygous pathogenic variant in *SALL1* associated with Townes-Brocks syndrome in both, the father and the daughter.

## Future direction

Due to the decrease in costs and advances in technology and bioinformatics, genome sequencing is likely to replace exome sequencing and panel diagnostics as the standard method for genetic testing in individuals with CAKUT in the coming years. The development of artificial intelligence in particular as well as the integration of multi-omics will be immensely helpful in improving the assessment of genetic variants. This, in turn, will bring about a lasting improvement in the clinical care of this patient group through personalized approaches.

Large-scale, global multi-omics studies in a translational setting, providing detailed (in-depth) phenotypic information from clinical patients with genetic, epigenetic, environmental, transcriptomic, proteomic, and metabolic data, will help to gain further insights into the development of these complex malformations. For example, a recent study has shown the added value of the integration of prenatal ultrasound findings as well as amniotic peptidome markers in the prediction of postnatal kidney function in CAKUT [29] leading to improvement of prenatal counselling and more accurate selection for genetic testing. Such promising efforts should be prospectively validated in larger cohorts before implementation into the clinical management of CAKUT.

## Conclusion

CAKUT encompasses a broad spectrum of developmental disorders of the kidneys and urinary tract, including extrarenal manifestations, caused by genetic and non-genetic factors. This phenotypic and genotypic heterogeneity requires individualized and multidisciplinary care for these patients from childhood to adulthood.

The integration of large-scale genomics with functional modeling has transformed CAKUT research from a descriptive to a mechanistic field. The next challenge lies in translating these discoveries into individualized diagnostic and therapeutic strategies.
